# Implication of NADPH Oxidases in the Early Inflammation Process Generated by Cystic Fibrosis Cells

**DOI:** 10.5402/2012/481432

**Published:** 2012-07-05

**Authors:** Nushjira Pongnimitprasert, Margarita Hurtado, Foudil Lamari, Jamel El Benna, Corinne Dupuy, Michèle Fay, Marie-José Foglietti, Maguy Bernard, Marie-Anne Gougerot-Pocidalo, Françoise Braut-Boucher

**Affiliations:** ^1^INSERM U-773, Centre de Recherche Biomédicale Bichat, Beaujon (CRB3), Faculté de Médecine Xavier Bichat, Université Paris Diderot Paris 7, 75018 Paris, France; ^2^Département de Biochimie, UFR des Sciences Pharmaceutiques et Biologiques, Université Paris Descartes, Sorbonne Paris Cité, 75006 Paris, France; ^3^Assistance Publique Hôpitaux de Paris, CIB Phenogen, Unité d'Immunologie Dysfonctionnement Immunitaire, Centre Hospitalier Universitaire Xavier Bichat, 75018 Paris, France; ^4^Centre National de la Recherche Scientifique, FRE2939, 94805 Villejuif Cedex, France

## Abstract

In cystic fibrosis (CF) patients, pulmonary inflammation is a major cause of morbidity and mortality. The aim of this study was to further investigate whether oxidative stress could be involved in the early inflammatory process associated with CF pathogenesis. We used a model of CFTR defective epithelial cell line (IB3-1) and its reconstituted CFTR control (S9) cell line cultured in various ionic conditions. This study showed that IB3-1 and S9 cells expressed the NADPH oxidases (NOXs) DUOX1/2 and NOX2 at the same level. Nevertheless, several parameters participating in oxidative stress (increased ROS production and apoptosis, decreased total thiol content) were observed in IB3-1 cells cultured in hypertonic environment as compared to S9 cells and were inhibited by diphenyleneiodonium (DPI), a well-known inhibitor of NOXs; besides, increased production of the proinflammatory cytokines IL-6 and IL-8 by IB3-1 cells was also inhibited by DPI as compared to S9 cells. Furthermore, calcium ionophore (A23187), which upregulates DUOX and NOX2 activities, strongly induced oxidative stress and IL-8 and IL-6 overexpression in IB3-1 cells. All these events were suppressed by DPI, supporting the involvement of NOXs in the oxidative stress, which can upregulate proinflammatory cytokine production by the airway CFTR-deficient cells and trigger early pulmonary inflammation in CF patients.

## 1. Introduction

Cystis fibrosis (CF) is a fatal autosomal genetic disorder caused by mutations in the cystic fibrosis transmembrane conductance regulator (CFTR) gene. The CFTR protein provides cAMP-regulated chloride conductance and acts as a regulator of apical Na^+^ absorption. Although many organs are affected in CF, lung disease is the major cause of morbidity and of virtually all mortality [1]. In CF, loss of CFTR function results in altered ion transport of the airway epithelium and rise in mucus viscosity. Increased mucus viscosity with reduced mucociliary clearance is a critical event in the susceptibility to bacterial infections [2–4]. A pathological feature of lung disease in CF is the presence of early and excessive inflammation [3, 5]. However, following the finding of neutrophil dominated inflammation in the absence of detectable bacterial or viral pathogens in bronchial lavages obtained from CF infants [6], the sequence of events at the onset of airway inflammation has been the subject of debates, hence, the proposal that inflammation could precede infection by some direct contribution of the defective CFTR [7, 8]. Furthermore, *in vitro* data have shown that lung epithelial cells, which express defective CFTR, produce high level of proinflammatory cytokines, especially IL-8 chemokine as compared to control cells [9–13]. This high IL-8 production can participate in the pulmonary neutrophil infiltration that occurs in CF.

In the CF disease, many factors can contribute to oxidative stress as the disease combines increased production of reactive oxygen species (ROS) and impaired antioxidant protection [5]. In CF a massive infiltration of neutrophils leads to the generation of ROS that are produced by the phagocytic NADPH oxidase and are largely responsible for patients' lung injuries. In addition, airway epithelial cells exhibit constitutive hydrogen peroxide production [14–16]. Recently, homologs of the catalytic subunit of the phagocytic NADPH oxidase (gp91phox, now renamed NOX2) have been identified in humans. They form the NADPH oxidase (NOX) family, which encompasses 7 members: NOX1, NOX2, NOX3, NOX4, NOX5, and Dual oxidases, (DUOX1, DUOX2) [17–19]. Besides their predominant location or initial discovery in some tissues (NOX2 was the prototypic NOX in phagocytes and DUOXs were initially identified in the thyroid tissue [19]) several NOX isoforms have been identified in different tissues [17]. In fact, DUOXs as well as NOX1, NOX2, and NOX5 have been found in airway epithelial cells [14, 15, 20, 21]. NOXs are dedicated to the production of reactive oxygen species (ROS), such as superoxide anions (O_2_^∙^^−^) and hydrogen peroxide (H_2_O_2_), by means of the single-electron reduction of molecular oxygen using NADPH as the electron donor [17].

ROS play a critical role in host defense against pathogens [22] and are also important mediators in cellular signaling [23, 24]. In particular, ROS are involved in cytokine synthesis, such as TNF, IL-1, IL-6, and IL-8, by phagocytes and epithelial cells [25–27]. Therefore, depending on the concentration, duration, and localization of their production, ROS either play a beneficial role by participating in tissue homeostasis or, when inappropriate or excessive production occurs, can directly damage surrounding tissues and participate in inflammatory disorders [22, 28]. In this context, oxidative stress has been shown to increase ERK1/2 MAP Kinase activity in CF lung epithelial cells, which could explain excessive IL-8 production by these cells [12]. However, the role and mechanisms of oxidative stress in early inflammation occurring in CF are not fully understood and arouse controversy. 

The aim of this study was to investigate the involvement of NOXs in the IB3-1 defective CFTR epithelial cell line in terms of oxidative stress and cytokine production in environmental conditions that could mimic CF conditions. Initial studies [29] suggested that the absence of CFTR led to a change in the composition of the airway surface fluid (ASL), namely an increase in NaCl concentration. In parallel [30], different data have supported a “low volume” hypothesis resulting in a decrease of periciliary fluid volume that leads to airway surface dehydration and mucociliary clearance decrease. As it is difficult to reproduce dehydration conditions *in vitro*, we used a model using various NaCl concentrations in accordance with previous studies that have shown that increased NaCl concentrations in the environment of CFTR defective human epithelial cells induced an increased production of cytokines such as IL-8 [31]. We hypothesized that ROS production depending on the modulation of NOX activity in CF cells could induce cytokine production participating to early inflammation in CF.

## 2. Materials and Methods

### 2.1. Materials

Eagle's Minimal essential medium (MEM), fetal calf serum (FCS), L-glutamine, penicillin, streptomycin, Hank's Balanced Salt Solution (HBSS) and phosphate buffered saline (PBS) were purchased from Gibco-BRL (InVitrogen, Cergy, France). Luminol, lucigenin, N-ethylmaleimide (NEM) and *tert*-butylhydroperoxide (*t*-BHP at 70% (v/v) in aqueous solution were purchased from Sigma Chemical Co. (St-Quentin-Fallavier, France). Diphenyleneiodonium (DPI), apocynin and calcium ionophore (A 23187) were obtained from Calbiochem (San Diego, CA, USA). 5′-chloromethyl fluorescein diacetate (CMF-DA), monobromobimane (MBrB), and Yopro-1 were purchased from Molecular Probes (Eugene, OR, USA). The horseradish peroxidase- (HRP-) conjugated goat anti-rabbit, goat anti-mouse, and the ECL western blot detection system came from Santa Cruz Biotechnology (Santa Cruz Biotechnology, Santa Cruz, California). The polyclonal rabbit antibody directed against the intracellular domain of human DUOX1/2 was a gift from Dr. C. Dupuy (Villejuif, France) [19]. The rabbit polyclonal antibodies directed against NOXA1 and NOXO1 were produced in the laboratory as well as the recombinant human proteins NOXA1 and NOXO1 [32]. The rabbit polyclonal antibodies against p47phox and p67phox were a gift from B. Babior. The rabbit polyclonal antibody anti-NOX1 was from Abcam; the monoclonal mouse anti-gp91phox antibody (54.1), the rabbit polyclonal anti-p22phox, and anti-NOX5 antibodies were from Santa Cruz Biotechnology; the rabbit polyclonal anti-NOX3 and anti-NOX4 antibodies were from Abnova and Novus Biologicals, respectively.

### 2.2. Cell Cultures

IB3-1 cell line (ECACC reference no. 88081201) (bronchial epithelial cells from cystic fibrosis patient immortalized by viral transformation using adeno-12-SV-40 with the CFTR genotype of Δ508/W1282X) and S9 cell line (ECACC reference no. 85011431) (corrected CFTR cells Δ508/W1282X + wild-type CFTR) generated from IB3-1 cells by transfection with a recombinant adeno-associated viral vector encoding full-length, wild-type CFTR were used; IB3-1 cells have the trafficking defect that characterizes primary CF cells. The IB3-1 and S9 clones were thoroughly characterized by many groups. Cells were cultured in MEM medium supplemented with 10% heat-inactivated FCS, antibiotics (100 *μ*g/mL streptomycin, 100 U/mL penicillin) and 2 mM L-glutamine and cultured in a humidified atmosphere containing 5% CO_2_ at 37°C. All cell cultures were negative for mycoplasm contamination (Mycoalert, Cambrex). The cells were cultured either into 75 cm^2^ tissue culture flasks at the initial concentration of 3.10^5^ cells/mL or in 96-well flat bottom microplates at 3.10^4^ cells/well. For these experiments, both cell types were grown near confluence (80%). For cytokine release, 10^5^ cells were plated and grown overnight in 300 *μ*L medium in 24-well plates. Cell viability was assessed using the Alamar blue assay [33]. Only cell populations with at least 95% viability were used for the experiments.

### 2.3. Exposure of Cystic Fibrosis Cells (IB3-1) and Corrected Cells (S9) to the Different Concentrations of NaCl in Extracellular Media

Cells were exposed to MEM without phenol red adjusted with NaCl solutions to obtain hypotonic, isotonic, or hypertonic media, respectively [10]. Ionic concentrations were calibrated with a pH/Ionometer (Radiometer Analytical, Hach Lange GMBH). For all cases pH was fixed at 7.4. The IB3-1 and S9 cells were cultured until sub-confluence in a complete MEM medium and incubated at 37°C, 5% CO_2_, and 95% air to ensure cells would adhere and proliferate. After that, subconfluent monolayers of IB3-1 and S9 cells were exposed to the three NaCl-containing solutions used in this study at pH 7.4 for different time periods according to the analyzed parameters. In some experiments, *tert*-butyl hydroperoxide (*t*-BHP), N-ethylmaleimide (NEM), diphenyliodonium (DPI), apocynin, or calcium ionophore (A 23187) were added to the cultures. Prior studies (time and concentration relationship) were carried out to determine the optimal concentrations required in terms of activity and viability. In all experiments, especially using DPI, cell viability was checked by Alamar blue assay.

### 2.4. Monitoring Oxidative Stress

Oxidative stress depends on an imbalance between ROS production and antioxidant systems. To assess the oxidative stress induced by the different culture media in IB3-1 and S9 cells, we measured ROS production intra- and extracellularly and the levels of glutathione, a major antioxidant compound, as well as total thiol content. To assess the source of ROS generated in the different experimental conditions, IB3-1 cells and S9 cells were preincubated for 15 min with DPI (2.5 *μ*M), which is an inhibitor of flavoproteins, namely, the catalytic subunit of NADPH oxidase. ROS production was measured using the chemiluminescence method currently used in the laboratory [34]. Briefly, cell suspension (10^6^ cells in 500 *μ*L HBSS) was preincubated without or with 2.5 *μ*M DPI or 200 *μ*M apocynin for 15 min, then 10 *μ*M luminol and 5 U/mL peroxidase were added to react in the thermostated chamber (37°C) of the luminometer (Berthold-Biolumat LB937) and allowed to stabilize. After a baseline reading was established, cells were incubated with the different NaCl media. Changes in chemiluminescence were measured over a 2-hour period in accordance with previous kinetic experiments. Results were expressed in terms of area under the curve (cpm/10^6^ cells) and the percentage between hypotonic and hypertonic treatment as compared to isotonic medium was calculated. 

Glutathione and thiol level measurements were performed using the 5′-chloromethyl-fluorescein diacetate (CMF-DA, 10 *μ*M) and the monobromobimane (MBrB, 40 *μ*M) probes, respectively. CMF-DA diffuses into the cells and is hydrolyzed into the fluorogenic CMF. The glutathione transferase catalyses the reaction between CMF and the intracellular thiols retained inside the cell, whereas the unconjugated probe is released in the medium and eliminated. A decrease in fluorescence reveals a decrease in GSH content. The monobromobimane probe, which forms a fluorescent adduct with sulfhydryl groups, allows measuring total thiols [33, 35]. After 30 min of incubation at 37°C, fluorescence intensity was measured at *λ*ex 485 nm and *λ*em 538 nm (Fluostar, BMG LabTechnologies, Champigny-sur-Marne, France). Reduced glutathione (GSH) or total thiol contents were expressed as the percentage of fluorescence intensity in each cell type incubated with hypotonic and hypertonic treatment as compared to isotonic medium. All measurements were done in triplicate. Experimental control thiol depletion was obtained by treatment of cells with the alkylating reagent N-ethylmaleimide (NEM), used at 0.2 mM for 15 min.

### 2.5. Measurement of IB3-1 and S9 Apoptosis

Cells cultured in 96-well plates were labeled with fluorogenic yopro-1 (20 *μ*M) probe. In these conditions, viable cells excluded yopro-1 whereas apoptotic cells took up the probe by intercalating into DNA. Microscopic examination was performed and fluorescence intensity was measured using Fluostar (BMG LabTechnologies, Champigny-sur-Marne, France) at *λ*ex 485 nm and *λ*em 538 nm, which are optimum wavelengths for yopro-1 [36]. Results were expressed as the percentage of fluorescence intensity in each cell type incubated with hypotonic and hypertonic treatment as compared to isotonic medium. 

The mitochondrial apoptotic pathway was detected as a loss of the mitochondrial transmembrane potential (ΔΨ_m_), measured as a reduction of the DiOC6 incorporation [37]. The cells, treated or not with the direct oxidant *t*-BHP (2 mM), used as a control of induced oxidative stress or NaCl media were loaded with DiOC6 (40 nM) for 15 min at 37°C. Then, they were incubated for 15 min with 1 mg/mL Propidium iodide to gate out dead cells. Samples were then suspended in PBS and analyzed immediately by flow cytometry (Beckman Coulter). Results were expressed in percentage of DiOC6^low^ cells.

### 2.6. Cytokine Quantification

IL-6 and IL-8 were measured using BD Cytometric Bead Array soluble protein Flex Set assays (Beckton Dickinson, BD Biosciences, Europe) by flow cytometric analysis. The secretion of IL-6 and IL-8 in the culture supernatants of IB3-1 and S9 bronchial epithelial cells was measured 18 hours after treatment with the different extracellular NaCl solutions in 24-well plastic tissue culture plates (10^5^ cells/well). Supernatants were then collected from each well and stored at −80°C. Flow cytometric analysis was performed with a Becton Dickinson FACSCanto II Immunocytometry System (BD Biosciences). The data were analyzed using the FCAP Array software (Becton Dikinson) and expressed as pg/mL using the standard Flex Set concentration curve. 

### 2.7. Western Blotting for NOX Isoforms and Regulatory Subunits

To keep the same cell density as in other experiments, 10^6^ cells of S9 or IB3-1 were plated in 25 cm^2^ polystyrene flask with complete MEM. In preliminary experiments, we tested DUOX1/2 and NOX2 expression after 5 h and 18 h of incubation. In our experimental model, the optimal expression of these proteins was obtained after 18 h of incubation. After the different extracellular NaCl treatments, the cells were washed with PBS Ca^2+^/Mg^2+^ free and scrapped in presence of a cocktail of proteinase inhibitors and fractionated under reducing conditions. Protein determination was carried out after sonicating each sample in ice by using Bio-Rad assay and 0.1 mg/mL BSA as standard. Samples containing 60 *μ*g of each extract from S9 and IB3-1 cells incubated with the different NaCl media were separated by 8% SDS-PAGE and blotted onto nitrocellulose membrane. The membranes were then blocked at room temperature for 1 hour with 5% milk prepared in 25 mM Tris Cl, pH 7.5, 150 mM NaCl (TBS), and containing 0.05% Tween-20 (TBS-T). The membranes were incubated overnight at 4°C with the antibodies directed against NOX1, its regulators NOXO1 and NOXA1, NOX2, its regulators p47phox and p67phox, NOX3, NOX4, NOX5, DUOX1/2 and p22phox at previously determined concentrations. After three washes in 0.05% TBS-Tween, the incubation with the secondary antibody (HRP-goat anti-rabbit or HRP-goat anti-mouse) was performed for 1 hour in blocking buffer. After washing revelation was performed by enhanced chemiluminescence. *β*-actin was used as a loading control. 

### 2.8. Statistical Analysis

All results were expressed as mean ± SEM. Significant differences were identified using a Student's *t*-test. Values of *P* inferior to 0.05 were considered statistically significant.

## 3. Results

### 3.1. Differential Oxidative Stress Responses of CF Cells and Non-CF Cells Are Suppressed by Diphenyliodonium

**Figure 1:**
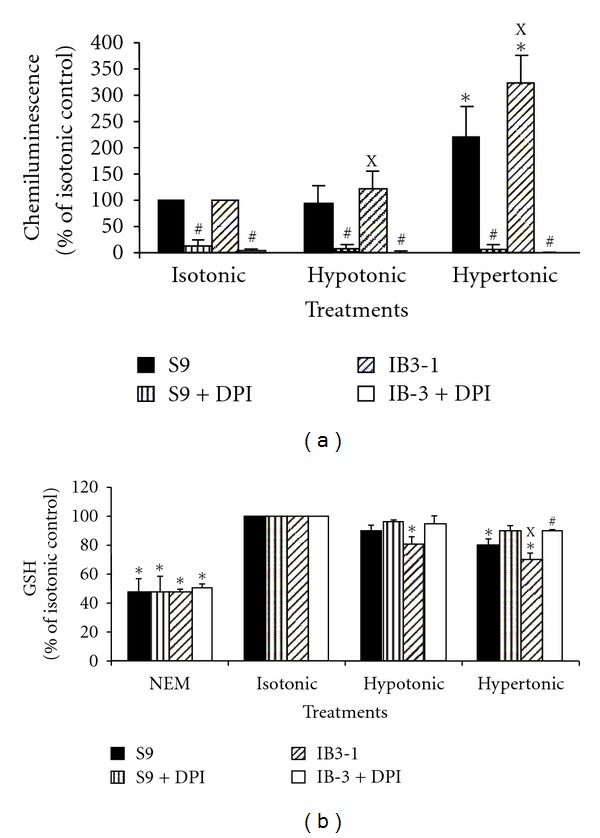
Oxidative stress in IB3-1 defective CFTR cells as compared with S9 control cells. Cells were treated or not by DPI (2.5 *μ*M) before incubation with the isotonic, hypotonic, or hypertonic media (a) ROS production was measured using a luminol-amplified chemiluminescence method as described in Section 2. Results were obtained in cpm/10^6^ cells/2 h and were expressed as the percentage of chemiluminescence intensity obtained for each cell type in hypotonic and hypertonic conditions as compared to isotonic conditions. Chemiluminescence of IB3-1 and S9 cells incubated in isotonic conditions was (11.3 ± 1.5) × 10^6^ and (11.4 ± 1.2) × 10^6^, respectively. (b) GSH content of IB3-1 defective CFTR cells and S9 control cells was measured as described in Section 2. NEM, which blocks sulfhydryl groups, was used as control. Results were obtained in arbitrary fluorescence units (AFUs) and were expressed as the percentage of AFUs obtained for each cell type in hypotonic and hypertonic conditions as compared with that obtained in isotonic conditions. AFUs of IB3-1 and S9 cells incubated in isotonic conditions were 34855 ± 1320 and 38870 ± 1637, respectively. Results are mean ± SEM (*n* = 3). ^X^IB3-1 significantly different from S9 in the same conditions (*P* ≤ 0.05); *significantly different from IB3-1 or S9 in isotonic medium, respectively (*P* ≤ 0.05); ^#^significantly different from nontreated with DPI in the same medium (*P* ≤ 0.05 ).

ROS production was monitored by luminol-amplified chemiluminescence as described in Section 2. In addition, the cells were pretreated or not for 15 min with 2.5 *μ*M DPI, an NADPH oxidase inhibitor. As shown in Figure 1(a), both airway epithelial cell lines IB3-1 and S9 show basal production of ROS in isotonic conditions. There was no significant difference between both cell lines (mean ± SEM = 11.4 × 10^6^  ± 1.2 × 10^6^ cpm/10^6^ cells/2 h and 11.3 × 10^6^  ± 1.5 × 10^6^ cpm/10^6^ cells/2 h for S9 and IB3-1, resp., *n* = 5). When treated with hypertonic medium, both cell lines increased their ROS production approximately two- and three-fold as compared to isotonic medium for S9 and IB3-1, respectively. IB3-1 ROS production was significantly higher than that obtained with S9 cells, *P* < 0.05, *n* = 5. Hypotonic medium also slightly increased ROS production by IB3-1 as compared to S9 cells whose production did not differ from that obtained in isotonic medium. The pretreatment with DPI revealed strong inhibition of ROS production that could suggest the implication of an NADPH oxidase in this process. Similar inhibition was obtained using apocynin (data not shown). To determine the role of thiols in cell defense against ROS production, the effects of ionic variations on GSH and total thiol contents after 2-hour-incubation time on S9 and IB3-1 cell lines were assessed using the CMF-DA (Figure 1(b)) and the monobromobimane fluorescent probes, respectively. As shown in Figure 1(b), hypo- and hypertonic media significantly decreased GSH intracellular content in the IB3-1 cell line as compared to IB3-1 cells maintained in isotonic conditions (100%). In hypertonic conditions, GSH content was significantly lower in IB3-1 cells than in S9 cells. Noteworthy, hypertonic medium also induced increased ROS generation and reduced GSH content in the control S9 cells. However, the hypertonic medium-induced alteration was significantly higher in the CFTR-deficient IB3-1 cells than in control S9 cells. These results are in accordance with Chan et al. who also showed an increased response of control cells to high salt concentrations [31]. 

 NEM treatment used as a common control to block sulfhydryls groups [33] dramatically decreased GSH in both cell lines, as expected. Hypertonic conditions induced similar variations on the total thiol content although no significant decrease was observed between IB3-1 and S9 cells (data not shown). Treatment with DPI suppressed the differences in GSH content induced by hypertonic medium (Figure 1(b)). Similar results were obtained using apocynin treatment (data not shown).

Altogether, variations in NaCl concentrations, especially the hypertonic conditions, induced a significant increase in ROS production associated with a tendency to decrease in GSH and thiol contents in IB3-1 as compared to S9 cell lines. These results suggest that hypertonic conditions induced a more important oxidative stress in defective CFTR IB3-1 cells than in control S9 cells. A pre-treatment with the DPI inhibitor suppressed these modulations, thus suggesting the involvement of NOX(s) in the induction of oxidative stress. Cell viability of both cell lines was checked to be higher than 90% by Alamar blue assay in all experimental conditions as indicated in Section 2.

### 3.2. Study of IB3-1 Cell Apoptosis as Compared with S9 Cells

**Figure 2:**
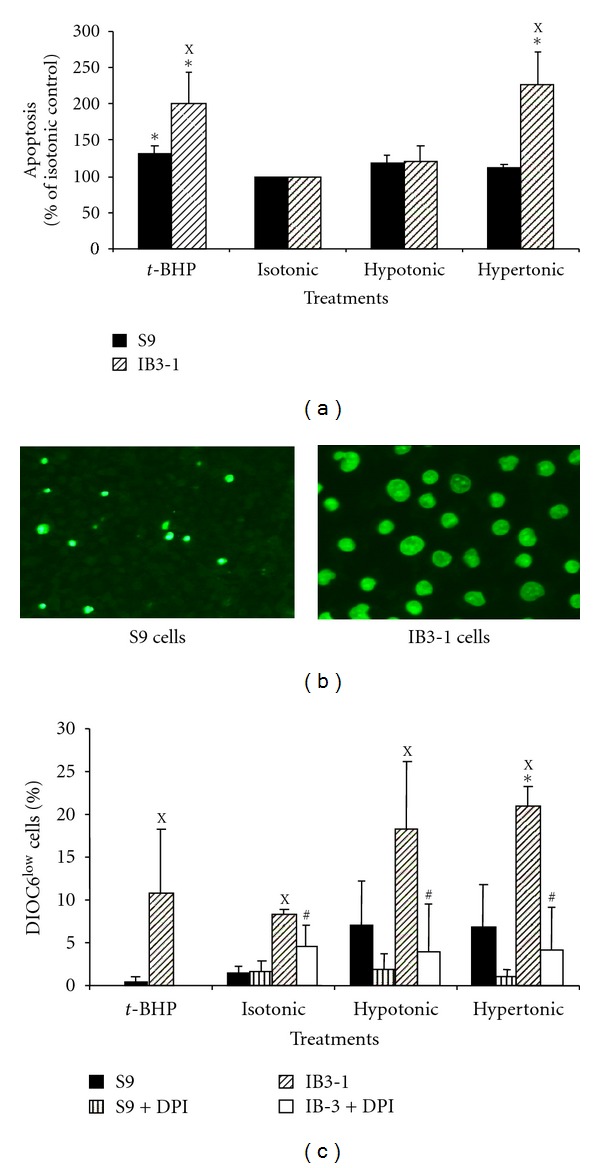
Apoptosis of IB3-1defective CFTR cells as compared with S9 control cells. (a) Cells were plated on 96-well microplates and treated by the different isotonic, hypotonic, or hypertonic media, then incubated for 5 h at 37°C, and apoptosis was measured using yopro-1 probe as described in Section 2. Results were obtained in arbitrary fluorescence units (AFUs) and were expressed as the percentage of fluorescence signal obtained for each cell type in hypotonic and hypertonic conditions as compared with that obtained in isotonic conditions. AFUs of IB3-1 and S9 cells incubated in isotonic conditions were 25664 ± 807 and 27857 ± 980, respectively. *t*-butyl hydroperoxide (*t*-BHP, 2 mM) was used as an oxidative stress inducer. Results are mean ± SEM (*n* = 3). ^X^IB3-1 significantly different from S9 (*P* ≤ 0.05); *significantly different from IB3-1 or S9 in isotonic medium, respectively (*P* ≤ 0.05). (b) Microscopic examination of IB3-1 and S9 after exposure to hypertonic medium for 5 h, then loaded with 10 *μ*M Yopro-1. Cells were examined in fluorescence microscopy (inverted microscope Olympus IMT2) (magnification ×300). (c) The mitochondrial apoptotic pathway was detected as a reduction of DiOC6 incorporation in mitochondrial membranes after incubation of IB3-1 cells and S9 cells in in the various ionic conditions for 5 hours, in the presence or absence of 2.5 *μ*M diphenyleneiodonium (DPI), as described in Section 2. *t*-butyl hydroperoxide (*t*-BHP, 2 mM), used as a control of oxidative stress, was incubated for 1 hour with both cell lines. Then cells were loaded with 40 *μ*M DiOC6 and further incubated for additional 15 min at 37°C. Cells were analysed by flow cytometry, and retention of DIOC6 was expressed in percentage of DiOC6^low^ cells. Values are means ± SEM (*n* = 3). ^X^IB3-1 significantly different from S9 in the same conditions (*P* ≤ 0.05); *significantly different from IB3-1 or S9 in isotonic medium, respectively (*P* ≤ 0.05); ^#^significantly different from non treated with DPI in the same medium (*P* ≤ 0.05 ).

As oxidative stress has been described to induce or accelerate cell apoptosis, we tested the effect of ionic variations in extracellular medium on the apoptosis of IB3-1 cells as compared to that of S9 cells. We used the fluorescent probe yopro-1, as described in Section 2, to test the rate of apoptosis of both cell lines in these conditions. The level of apoptosis in isotonic conditions was low (<5% of the cells) and no difference between S9 and IB3-1 epithelial cells was noted (Figure 2(a)). In contrast, culture for five hours in hypertonic medium induced a higher level of fluorescence of the majority of IB3-1 cells as compared to S9 cells (Figures 2(a) and 2(b)). In hypertonic conditions apoptosis level of S9 cells did not significantly differ from that observed in isotonic conditions (Figure 2(a)). Treatment with *t*-BHP used as a control of oxidative stress [33] also showed that IB3-1 cells are significantly more sensitive to oxidant-induced apoptosis than S9 cells.

To further identify this hypertonic-induced apoptosis of IB3-1 cells, we measured the effects of the different NaCl containing media on mitochondrial apoptotic pathway by measuring ΔΨ_m_. The proportion of DiOC6^low^ (loss of ΔΨ_m_) IB3-1 cells was significantly higher than that of S9 cells after culture in hypertonic and hypotonic media, as compared to the value obtained in isotonic conditions (Figure 2(c)). Furthermore, the proportion of DiOC6^low^ IB3-1 cells was also significantly higher than that of S9 cells at the basal level in isotonic condition. The differences obtained between Yopro-1 and DiOC6 techniques in isotonic medium could be explained by a higher sensitivity of reduction of the DIOC6 incorporation in relation to the loss of mitochondrial transmembrane potential. These results suggest that apoptosis was triggered, at least in part, through an effect at/or upstream the mitochondrial membrane permeabilization. The increase percentage in DiOC6^low^ IB3-1 cells observed in all ionic conditions was significantly inhibited by DPI, thus reinforcing the hypothesis of the involvement of NOX(s) in the induction of apoptosis via ROS production.

### 3.3. Increased Cytokine Production by IB3-1 Cells Is Inhibited by DPI

**Figure 3:**
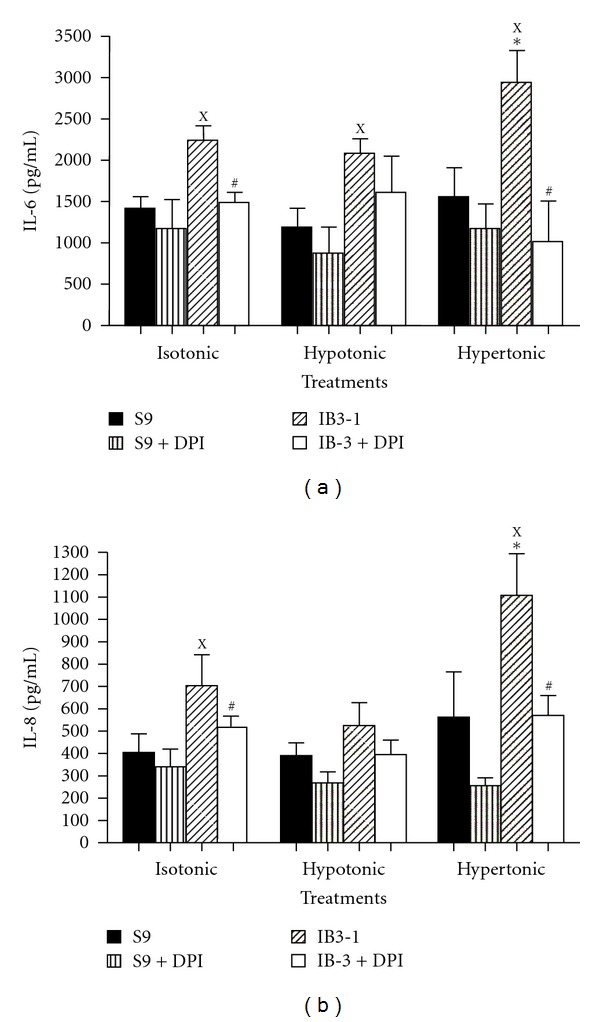
IL-6 and IL-8 productions by IB3-1 defective CFTR cells as compared with S9 control cells. Cells were cultured in 24-well plates and treated with various ionic conditions for 18 h in presence or absence of DPI. (a) IL-6 and (b) IL-8 measurements were performed in the supernatants as described in Section 2. Values are means ± SEM from three different experiments done in triplicate. ^X^IB3-1 significantly different from S9 in the same conditions (*P* ≤ 0.05); *significantly different from IB3-1 or S9 in isotonic medium, respectively (*P* ≤ 0.05); ^#^significantly different from nontreated with DPI in the same medium (*P* ≤ 0.05).

To evaluate the effect of NaCl treatments on IL-6 and IL-8 release, S9 and IB3-1 cell supernatants were harvested 18 hours after exposure to the various ionic concentrations media. The results presented in Figures 3(a) and 3(b), confirmed previous data showing an increased basal production of IL-6 and IL-8 by IB3-1 cells as compared to S9 cells that was further increased by hypertonic media. IL-6 production was also increased by hypotonic medium. DPI significantly inhibited basal as well as hypertonic-induced IL-6 and IL-8 increased production by IB3-1 cells (Figures 3(a) and 3(b)). 

### 3.4. NOX Protein Expression in CF Cells

**Figure 4:**
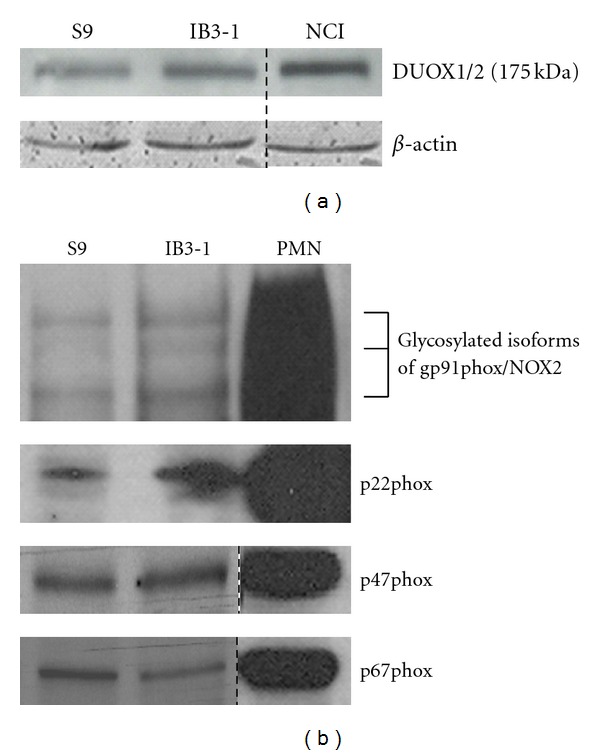
DUOX1/2 and NOX2 proteins expression in IB3-1 defective CFTR cells and in S9 control cells. DUOX1/2 were detected with an apparent molecular weight of 175 kDa by SDS PAGE and immunoblotting using a rabbit polyclonal antibody directed against human DUOX1/2 as described in Section 2. The human lung mucoepidermoid carcinoma derived cell line NCI-H292 was used as control for DUOX1 and DUOX2 positive cells. Neutrophils isolated from human blood were used as controls for NOX2 (gp91phox), p22phox, p47phox, and p67phox expression, which were detected by SDS-PAGE and immunoblotting using antibodies described in Section 2. *β*-actin was used as a loading control. (a) Basal expression of DUOX1/2 in IB3-1 and S9 cells cultured in isotonic medium. (b) Basal expression of gp91phox (NOX2), p22phox, p47phox, and p67phox in IB3-1 and S9 cells. Representative of three experiments. Dotted lines indicate that intervening lanes have been spliced out.

As DPI significantly inhibits oxidative stress, increased apoptosis as well as increased IL-8 and IL-6 production in CFTR-deficient cells cultured in hypertonic medium but also at the basal level for apoptosis and cytokine production, we examined the NOX protein expression that was a major source of ROS in airway epithelial cells. Using antibodies which recognize NOX1, its regulators NOXO1 and NOXA1, NOX2, its regulators p47phox and p67phox, NOX3, NOX4, NOX5, DUOX1/2, and p22phox, we showed by SDS/PAGE and western blot analysis that in basal isotonic conditions IB3-1 and S9 cells expressed DUOX1/2 as well as gp91phox (NOX2) and its partners p47phox, p67phox and the widely expressed p22phox (Figures 4(a) and 4(b)). The human lung mucoepidermoid carcinoma derived cell line NCI-H292 (CRL 1848; American type Culture Collection (ATCC), Manassas, VA) known to express DUOXs was used as positive control for DUOX1/2 testing [38] and lysates from neutrophils isolated from human blood, as usually performed in the laboratory, were used as controls for phagocytic NADPH oxidase compounds: NOX2, p22phox, p47phox, and p67phox [39, 40]. NOX1, NOXO1, NOXA1, NOX3, NOX4, and NOX5 could not be detected in both cell lines as compared to cell lines expressing these NOXs and recombinant proteins NOXO1 and NOXA1 produced in the laboratory (data not shown). The expression level of DUOXs, NOX2, and its partners was not significantly different between S9 and IB3-1 cells. 

After culture for 18 hours in presence of media containing various NaCl concentrations, DUOX1/2 and NOX2 protein expression was not modulated by ionic variations (data not shown). This did not preclude any ionic variation-induced enhanced activity.

### 3.5. Modulation by Calcium Ionophore

**Figure 5:**
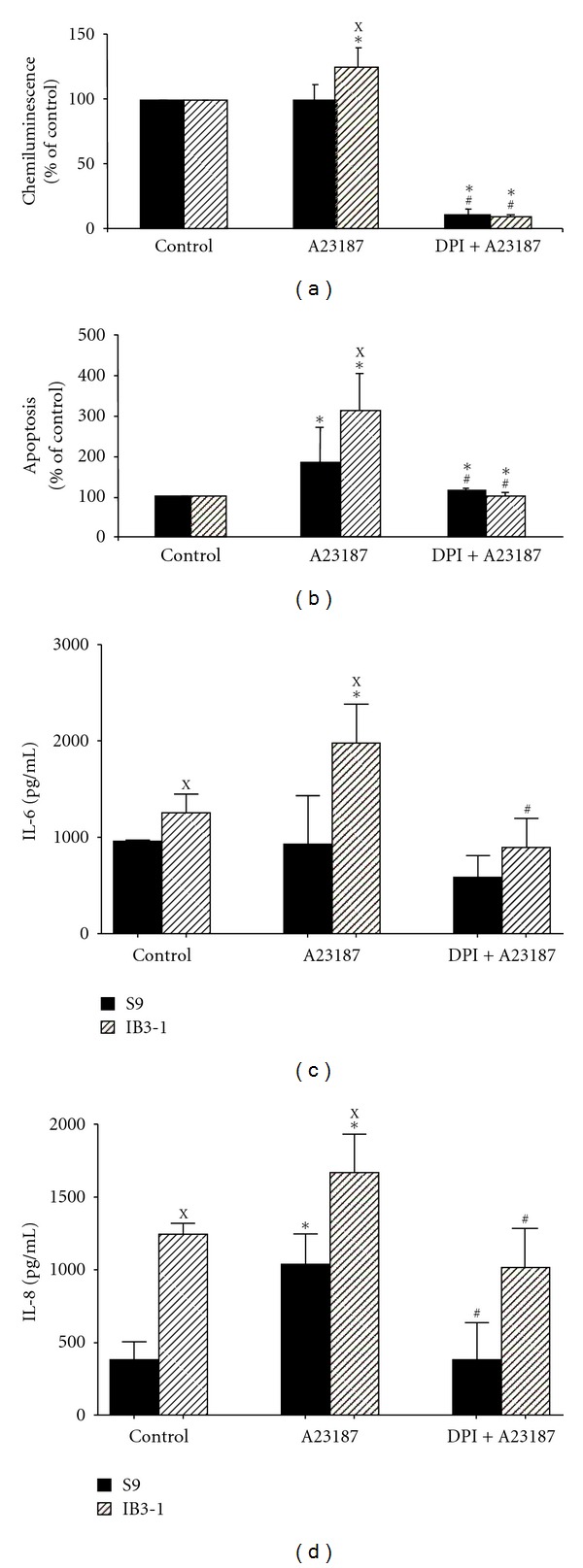
Effect of calcium ionophore A23187 in presence or absence of diphenyleneiodonium (DPI) on ROS production, induction of apoptosis, and IL-6 and IL-8 production by S9 and IB3-1 cells. S9 and IB3-1 cells were incubated in isotonic culture medium alone (control) or in medium with 5 *μ*M calcium ionophore in presence (A23187 + DPI) or absence (A23187) of 2.5 *μ*M DPI. (a) Luminol-amplified chemiluminescence. Results were expressed in relative ratio to control S9 or IB3-1 cells. (b) Apoptosis was measured using Yopro-1 as described in Section 2. Results were expressed in relative ratio to control S9 or IB3-1 cells. (c) IL-6 and (d) IL-8 production by S9 and IB3-1 cells as described in Section 2. Results were expressed as pg/mL. Results are expressed as mean ± SEM, *n* = 3. ^X^IB3-1 significantly different from S9 in the same conditions (*P* ≤ 0.05); *significantly different from IB3-1 or S9 in control isotonic medium, respectively (*P* ≤ 0.05); ^#^significantly different from A23187 alone (*P* ≤ 0.05).

The intracellular presence of two EF-hand motifs in DUOX proteins suggests that calcium may directly regulate these enzymes, which is consistent with the observations showing that calcium ionophore stimulates ROS production in thyroid and airway epithelial cells [15, 41, 42]. In addition, calcium regulates phagocytic NADPH oxidase activity [43]. As calcium homeostasis is altered in CF cells [44] and could be implicated in DUOXs and NOX2 regulation [20], we tested the effect of calcium ionophore (A23187) on the oxidative stress markers of both S9 and IB3-1 cells as compared to their respective control in isotonic conditions. A23187 significantly increased ROS production, apoptosis commitment, and IL-6 and IL-8 production by IB3-1 cells as compared to the untreated IB3-1 cells and to A23187-treated S9 cells (Figures 5(a), 5(b), 5(c), and 5(d)). In addition, the A23187-increased parameters were strongly suppressed by DPI in IB3-1 cells (Figures 5(a), 5(b), 5(c), and 5(d)). 

## 4. Discussion

This study showed that diphenyleneiodonium, a commonly used inhibitor of flavoenzymes such as NOXs, inhibited several parameters known as participating in oxidative stress in IB3-1 defective CFTR epithelial cells cultured in hypertonic environment. In fact, increased ROS production and decreased antioxidant component level such as reduced glutathione (GSH) and total thiol contents, which were altered in IB3-1 defective CFTR epithelial cells cultured in hypertonic environment, were inhibited by pretreatment of the cells with DPI. These results were confirmed using apocynin known as NOXs inhibitor and radical scavenger [45]. In addition, the increased involvement of the mitochondrial apoptotic pathway which was observed in IB3-1 defective CFTR epithelial cells cultured in basal isotonic conditions as well as in hypotonic and hypertonic conditions as compared to the S9 control cells in the similar conditions was also suppressed by DPI. Furthermore, calcium ionophore (A23187), which is known to upregulate some NADPH oxidase activities, strongly enhanced ROS production by IB3-1 cells and triggered their apoptosis as well as their IL-6 and IL-8 production and these events were suppressed by pretreatment of the cells with diphenyleneiodonium. The increased IL-8 and IL-6 production by defective CFTR epithelial cells in basal conditions, which has already been described [10, 11, 31], was also inhibited by DPI. The inhibitory effect of DPI supports the involvement of NADPH oxidases in all these events occurring in CF airway epithelial cells. We therefore investigated the presence of the different NOX isoforms in both cell lines. The results showed the expression of DUOX protein as well as NOX2 and its classical phagocytic partners. Variations of ionic conditions in the culture medium did not significantly modify the NOX expression. However, these results did not preclude any ionic variation-induced NOX activity. These results suggest the intrinsic role of CFTR deficiency in inducing oxidative stress and increased cytokine production via NOX activity leading to early inflammatory events in the CF disease.

The dysfunction of CFTR leads to increased mucus viscosity via periciliary liquid layer depletion and therefore abnormal environment of the epithelial airway cells. As it was difficult to reproduce these pathologic features* in vitro*, we chose the model of ionic variations in relation to various concentrations of NaCl as a stress model of the cells. In fact, the alterations of the different parameters tested were often modified in hypotonic as well as in hypertonic conditions. 

We showed here for the first time that IB3-1 defective CFTR cells express DUOXs and NOX2. We used antibody directed against the intracellular domain of DUOX1 and 2 (a gift from C. Dupuy), as commercial antibodies against DUOX1 and DUOX2 did not give accurate results. Recent works have demonstrated the presence of DUOX proteins in the respiratory tract epithelium and have shown that DUOX proteins are an important source of ROS production in the respiratory tract [14, 15]. In addition, other NOX isoforms have been detected in aiway epithelial cells such as NOX1, NOX2, and NOX5 [20, 21]. Investigating by immunoblotting the presence of the 5 other NOXs only showed the presence of NOX2, its regulators p47phox and p67phox, and the widely distributed p22phox. Interestingly, calcium ionophore A23187 significantly enhanced ROS production, apoptosis, and cytokine production by IB3-1 cells, as compared to untreated cells, as well as A23187-treated S9 cells. Previous works have shown that calcium homeostasis is altered in cells presenting CFTR mutations [44]. Furthermore, studies have linked altered intracellular calcium mobilization, NF-*κ*B activation with inflammatory response in several cell systems in CF [5, 46]. In particular, an increase in IL-8 secretion in CF cells was mediated by an increased intracellular calcium mobilization [3]. These events are in accordance with the presence in IB3-1 cells of both NADPH oxidases, DUOX1/2 and NOX2 whose activities are regulated by calcium ions [15, 17, 41, 43]. The A23187-induced upregulation of ROS production, apoptosis, and cytokine production by IB3-1 cells were strongly inhibited by DPI, suggesting a relationship between intrinsic defect in CFTR and NOX protein activity.

Our results suggest that IB3-1 defective CFTR cells are highly susceptible to ionic variations via DUOX and NOX2 activities generating ROS production. Increased ROS production and modulation in GSH epithelial transport as well as increased susceptibility to apoptosis have already been described in CFTR-deficient cells but their mechanism was unclear [2, 46]. Our data suggest the involvement of DUOX and NOX activities in these processes. Oxidative stress has been described to enhance cytokine production [23–26]. Our results confirm an increased production of the proinflammatory cytokines IL-6 and IL-8 by IB3-1 defective CFTR cells in basal isotonic medium, as well as in various ionic conditions, as already described [5, 10, 11]. We showed here that DPI significantly reduces this production, suggesting also the involvement of DUOX and NOX2 in this process and therefore in the early inflammatory status of the lung in CF patients. 

## 5. Conclusions

In conclusion, we have shown that inhibition of catalytic subunit of NADPH oxidases isoforms, that is, DUOX and NOX2 detected in IB3-1 defective CFTR cells, suppresses features of oxidative stress, which are observed in hypertonic conditions as well as the increased IL-6 and IL-8 production that could be responsible for neutrophil migration and early inflammation in the course of the CF disease. The relationship between CFTR deficiency and NOX activities remains to be identified.
